# GPS Week Number Rollover Timestamp Complement

**DOI:** 10.3390/s21237826

**Published:** 2021-11-24

**Authors:** Majdi K. Qabalin, Muawya Naser, Wafa M. Hawajreh, Saja Abu-Zaideh

**Affiliations:** 1 Department of Computer Science, Princess Sumaya University for Technology, 1438 Al-Jubaiha, Amman 11941, Jordan; M.Aldalaien@psut.edu.jo (M.N.); saj20208080@std.psut.edu.jo (S.A.-Z.); 2 Engineering Department, Traklink Co., Amman 11941, Jordan; Wafa@trak-link.com

**Keywords:** GPS rollover, global navigation satellite system, timestamp complement, week number, Global Positioning System, trigger

## Abstract

Global Positioning System (GPS) is a global navigation satellite system and the most common satellite system used in navigation and tracking devices. The phenomenon of week number rollover happened recently—a year ago—due to a design limitation in the week number variable that counting weeks which causes vast losses. As many fleet management systems depend on GPS raw data, such systems stopped working due to inaccurate data provided by GPS receivers. In this paper, we propose a technical and mathematical analysis for the GPS week number rollover phenomenon and suggest a solution to avoid the resulting damage to other subsystems that depend on the GPS device’s raw data. In addition, this paper seeks to provide precautionary measures to deal with the problem proactively. The Open Systems Interconnection model (OSI) and transport layer level solution that has been suggested depends on a TCP packet reforming tool that re-formats the value of the week number according to a mathematical model based on a timestamp complement. At the level of the database, a solution is also suggested which uses triggers. A hardware-level solution is suggested by applying a timestamp complement over the GPS internal controller. Complete testing is applied for all suggested solutions using actual data provided by Traklink—a leading company in navigation and fleet management solutions. After testing, it is evident that the transport layer level solution was the most effective in terms of speed, efficiency, accuracy, cost, and complexity. Applying a transport layer level complement mathematical model can fix the consequences of GPS week number rollover and provide stability to all subsystems that used GPS data from infected devices.

## 1. Introduction

Time data that is transmitted by operational satellites and used by the Global Navigation System (GPS) is mathematically referenced as the GPS until 1990 and GPS Time (GPST), based on the U.S. Naval Observatory (USNO) Atomic time scale. USNO is based on both International Atomic Time (TAI) and UTC, using periodic corrections. After 1990, time information was calculated using all operational stations and satellite clocks. Few microseconds can be accepted as a difference between the GPS and global time standard or the so-called Universal Time Coordinated (UTC), which is calculated by the International Bureau of Weights and Measures (BIMP) by applying a mathematical model that includes data from about 240 atomic clocks around the globe that maintain a local version of UTC [1].

BIPM achieves ultimate precision, by a few nanoseconds, using comparison processes. For this reason, leap seconds are added in calculations for UTC every specific period in order to maintain ultimate synchronization with the Earth’s rotational period concerning the sun. Such a process is necessary to obtain the proper correction for the time [2]. Due to the physics of the Earth’s rotation and some uncontrolled variables, we can see such differences; for example, the yearly average length of the day is currently about two milliseconds longer than it was 20 years ago [3]. GPS Time has no leap-seconds within the integer level. Leap-seconds added to UTC have affected GPS relational difference, as GPS time is always ahead of UTC by a few nanoseconds, and this value varies from day to day [4]. The corresponding precise GPS time accuracy is within 200 nanoseconds. While the Standard Positioning Service (SPS), a positioning and timing service, provides almost 340 nanoseconds of accuracy. The current GPS week segment is included in the sub-frame as one of the navigation messages that is considered part of the satellite clock, and other user information is accommodated to represent only 10 bits for the week value. Using 10 bits, the maximum number that can be represented is 210, which equals 1024, and thus we have a range of 0–1023 for week representation. As such, the GPS week number is modulo 1024, and at the end of the week 1023, the week counter will reset and rollover to zero; herein lies the problem. GPS time is based on modular arithmetic and is restricted to integers with a finite value that is defined; for example, in the 24-h time system, if it is currently 1800, then 8 h later, we say it is 0200, not 2600 [5].

Within the first version of the GPS interface control document ICD-GPS-200, a note stated: “At the expiration of GPS week number 1023, the GPS week number will rollover to zero (0)”. Users must account for the previous 1024 weeks, and this means that a GPS receiver should precisely determine reaching the maximum limit of week number by itself as the navigation message does not include year or week rollover information [6]. In this paper, we will use the timestamp format used in Unix as a base for complementing calculations according to UTC; within this mathematical model, we will take Greenwich Mean Time (GMT) as a reference time zone. It is important here to clarify that GMT is the denomination of a time zone, while UTC is the title of the time standard.

Table 1 clearly shows the difference between each occurrence by week 1024, which represent 19.7 years or 7168 days. In the fourth occurrence, the end of the cycle will occur on 6 January, 2137 as the more recent CNAV protocol, successor to the original NAV protocol uses thirteen-bit week numbers, 213, which can represent 8192 weeks, 0-8191 or 157 years, and as CNAV stated, the starting point from 1980 and after adding 8192 weeks to 1980 we will reach the next occurrence GPS rollover at 6 January, 2137. The effect of the GPS rollover problem on navigation and tracking device data can be seen in Table 2, which represent the data collected from different models of navigation devices using GPS modules [7].

As shown in Table 2, if we made a simple math process to count the weeks between the dates, we would find that each device has an incorrect date value that is equal to 1024 weeks. For example, the Bofan PT95 model reported 28 April 1991, while the correct date was 12 December 2010; subtracting two dates will lead to 7168 days or 1024 weeks. This example of corrupted data can cause significant damage to other subsystems which depend on GPS data. Fleet Management Systems (FMS) will stop functioning correctly due to the inaccurate raw data provided by GPS devices, as all of these data and synchronization is not valid. To handle such design limitations and the cascading effects of such issues, we need to understand that changes to the internal firmware of the GPS module are not possible except by the vendor, who completes this according to worldwide protocols, and in our case, packet size and form based on CNAV protocol.

Changing the firmware of the main controller used in GPS and fleet management devices is possible and much easier than previously, as we now have many vendors worldwide, and each of them has his own way of developing this kind of device, resulting in different programming models and structures being used [8]. For example, TELTONIKA Inc. devices—a well-known fleet management vendor that operates widely around the world—managed to build a new version of the firmware using a specific mathematical model to fix the wrong date that was interpreted by GPS modules. However, in such a case, much physical work is needed to bring to each device the specific tool that is used to upgrade the firmware. Over Air OTA programming is a good choice, in this case, which will minimize the operation cost in the upgrading process, but in many cases, it does take time for the vendor to apply such a tool as it requires significant of development and testing processes [9].

During the events of 6 April 2019, and after analyzing the problem, we designed a solution that provides an easy way to fix interpreted data before inserting the data into the database of a fleet management system. Our proposed solution also provided a reliable search mechanism that looks for previously processed data inside the database that may have caused a wrong calculation for subsystems. Unix epoch or timestamp is the base in our mathematical model calculations. Unix epoch, which is a system of describing a point of time, represents the number of seconds that have elapsed since the Unix epoch, minus the leap seconds. Using the Unix epoch timestamp representation as a starting point, and adding the GPS first epoch time with respect to the 1024-week differences, will finally determine the exact number of seconds that should be added to a rollover date and finally produce a correct value [10].

## 2. Materials and Methods

### 2.1. Mathematical Analysis

UTC and GPS time deviate (on average) every 18 months by one additional second. A leap second introduced in UTC documentation is necessary to adjust for changes in the Earth’s rotation; in our model, we use the average value for this deviation “Td”. The GPS CNAV message reports the offset between GPS time and UTC. As of January 2017, GPS time is 18 s more than UTC because of the leap second added to UTC on 31 December 2016. Most clocks derive their time from Coordinated Universal Time (UTC), however, the atomic clocks at the satellites are set to GPS time. The difference is that GPS time is not permanently corrected to the exact value of the rotation of the Earth, and as such it does not include leap seconds or other corrections which are periodically introduced to UTC. GPS time was set in order to have no offset with UTC in 1980; however, we have few correction manners that keep GPS time fixed at a steady offset with International Atomic Time. Periodic corrections are performed to the satellites’ clocks to keep them synchronized as much as possible with ground clocks [11].

UTC is a time standard based on Coordinated Universal Time, the mean solar time at the Royal Observatory in Greenwich, England. It was established in 1972 for civil purposes. UTC’s time of day can be expressed by the following formula: “UTC = (GMT + 0)/60 + DST”. This means that UTC is a scaled-down replica of Greenwich Mean Time.

This standard was adopted to simplify navigation and commerce by avoiding confusion about time zones and daylight-saving time. The offset from GMT has changed over the years; it was once as high as 17 h (17:00 UTC was equal to midnight GMT). Over the last century, it has been reduced to less than one hour (1:00 UTC = 18:00 GMT).

**Figure 1:**
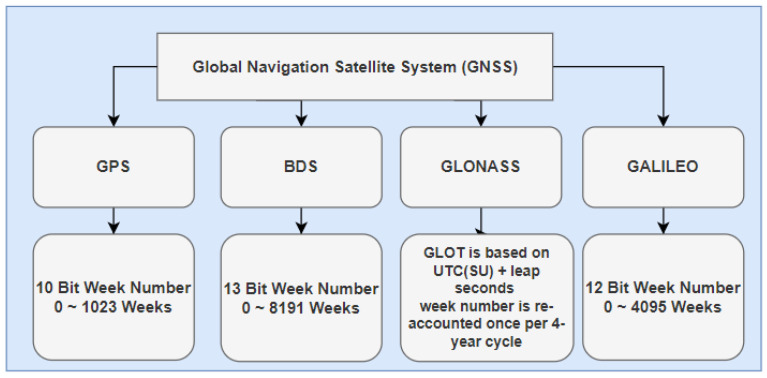
Week number ranges for different GNSS systems.

As shown in Figure 1, each GNSS system uses different limits for the week number value. The actual range for this value is purely an architecture issue. For GPS, and according to the CNAV message, the week number counter is limited, as 10 bits in binary representation can only represent an 1024 weeks. GPS week number limit is considered the smallest value among other GNSS systems, and so, by design, once the counter reaches the maximum value it will count down again to zero, as explained above. As shown in Table 3 we represent the mathematical symbols used in mathematical representation.

UTC and GPS time deviates (on average) every 18 months by one additional second. This is called a leap second, and has been introduced in UTC time documentations. It is necessary to adjust for changes in the Earth’s rotation. In our model we use the average value for this deviation “Td”.

(1) $$\mathrm{UGs}=\mathrm{Offset}\left(\mathrm{Gs}\right)-\mathrm{Offset}\left(\mathrm{Ut}\right),$$

(2) UGs=Ts (“6 January 1980”)−Ts (“1 January 1970”),

(3) $$\mathrm{Ws}\left(T\right)\;=\;\mathrm{Ws}\times 1024,$$

(4) $$\mathrm{OTs}=\mathrm{Ts}\;(\mathrm{STs})+\mathrm{Ws}+\frac{\mathrm{Td}}{\mathrm{UGs}},$$

As shown in Equation (1), the difference between the UNIX timestamp offset is calculated by subtracting the offset between the 80s and 70s epochs. We then have to calculate that offset in terms of 1024 weeks in order to use it later in total offset calculations on Equation (4), which will calculate the final value of the date after adding 1024 weeks and taking into consideration leap seconds for the whole 1024 weeks.

The GPS CNAV message report the offset between GPS time and UTC. As of January 2017, GPS time is 18 s more than UTC on account of the leap second that was added to UTC 31 December 2016. Most clocks derive their time from Coordinated Universal Time (UTC), but the atomic clocks at the satellites are set to GPS time. The difference is that GPS time is not always corrected to the exact value of the rotation of the Earth, and so it does not include leap seconds or other corrections which are periodically introduced to UTC. GPS time became set to have no offset with UTC in 1980. There are few correction manners that keep GPS time fixed at a steady offset with international Atomic Time. Periodic corrections are performed to the satellites clocks to keep them synchronized as much as possible with ground clocks [12].

A leap second is a second that is added to coordinate universal time (UTC) with solar time. Coordinated Universal Time (UTC) is the primary international standard for time, but it has no provision for leap seconds. The Earth’s rotation rate is not constant, and so, on average, UTC and solar time diverge by roughly one second per day. Leap seconds are a small, yet vital correction to the Earth’s timekeeping system. The Earth’s rotation slows slightly during each day and night, leading to a difference between the actual time and the standard astronomical time known as Universal Time Coordinate (UTC). The International Bureau of Weights and Measures (BIPM) periodically makes corrections to UTC by adding or subtracting leap seconds. On average this happens every 18 months with a total of 26 leap seconds added from 1972–2006. When UTC and GPS time deviates every 18 months by one additional second, it is called a leap second. This is done to ensure that the time on atomic clocks remains synchronized with the Earth’s natural phenomena. Jump seconds were first introduced in 1972 as a way of ensuring that atomic clocks would always be synchronized with the Earth’s natural phenomena, which is important for international trade, telecommunications, and other fields.

### 2.2. Theoretical Application

#### 2.2.1. Firmware

Firmware bugs are the unpopular guest inside the system that looms big in every developer’s mind. Firmware development issues can have catastrophic results for electronics devices, like navigation devices, and in many cases, fixing them incurs a significant cost, as they are often exceedingly hard to locate and attach in most cases. Usually, they are discovered by experience or through not taking some future time-related factors into account during development. The GPS week number issue is actually an overflow case. In computing, overflow mistakes can occur when a calculation is accomplished but the system is unable to handle the answer successfully. All computer systems have a predefined range of values that they can represent or store. Overflow mistakes occur when the execution of a set of instructions returns a fee outside of this variety and, in this case, the system normally provides us with wrong values. The GPS week number rollover is one of the cases in which a specific variable that was used to represent the weeks could not hold more than 1024 weeks, leading to a counter reset which resulted in the wrong raw data being used across all systems which depended on this variable, including the most important item, which is, in this case, the date [13].

**Figure 2:**
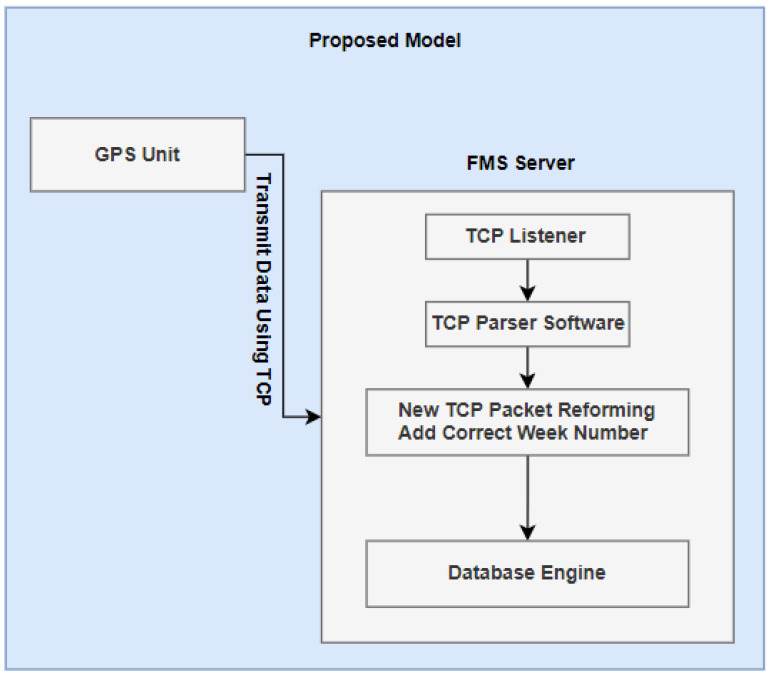
Path and stages of data transmission for GPS packets starting from GPS unit until data gathering inside the database system used as the base of all of the fleet management system operations according to our proposed model.

As shown in Figure 2, in the stages of data transmission, according to our proposed solution, the GPS week number problem was originally a firmware issue, and as such we can change the size of variable used to handle week number counting, but as the CNAV packet is limited from inside the satellites, we only can make updates on the GPS unit firmware. When the data reach stage two, this is purely a TCP/UDP socket. We also have the chance to modify data before it goes to the third stage between the database and network socket handler, and, finally, we could implement specific procedures over the database to fix the data, as needed. As such, the TCP Parser will receive the packet and apply mathematical equations and then transmit the data to the database engine. In this case, the week number data will have been corrected inside the database and FMS system will function properly.

Firmware updates normally take time and incur costs, as such operations include electronics and programming experience in which complexity level of firmware update will make finding such a solution much more difficult, even in devices for which vendors update the firmware, there is a long list of processes and procedures included that are required to guarantee the stability for the new firmware and to make sure that this firmware will not affect other parts of the device’s operations. Meanwhile if the device is not provided with the Over-The-Air (OTA) firmware upgrade infrastructure, the cost and complexity will be much higher, as such operations require a technical team to handle each device manually and connect it to a special equipment to upgrade the firmware.

**Figure 3:**
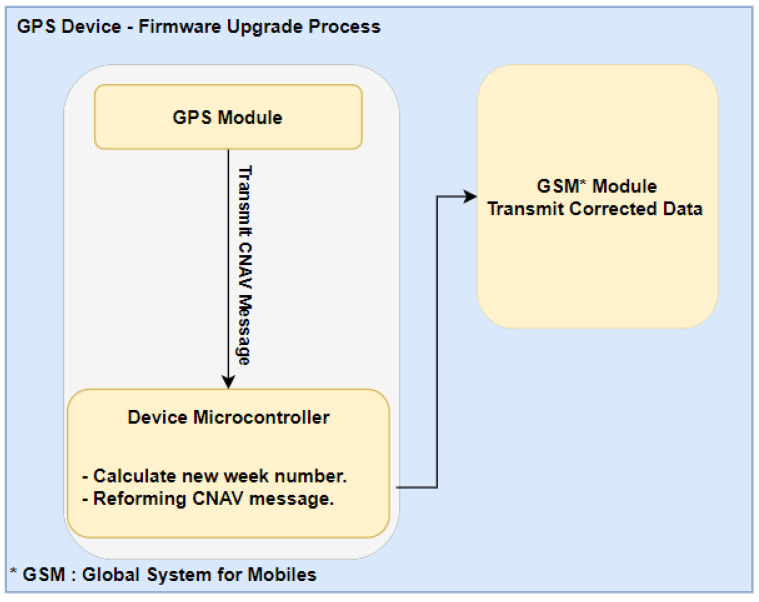
Firmware Upgrade Process.

As shown in Figure 3, normally Firmware updates to address such issues are easily solved in terms of coding and programming but it is hard to apply such solutions over all legacy devices that were made before such upgrades or patches were issued. The cost and operational effort needed are high and not easy to apply, even with OTA enabled devices there are still operational costs involved in uploading new patch to all old devices.

#### 2.2.2. Device Packet Protocol Handler

Most of the GPS devices that are used for a variety of based-systems, such as navigation, steering systems or even drone applications, use the National Marine Electronics Association (NMEA) which is the worldwide standard used for GPS data representation. Using a specific packet handler working over network presentation layers will rephrase the NMEA packet and ensure the proper updates that are needed to fix GPS week number value before parsing the data into a database handler. Such a solution will cause a backpressure problem in the data tunnel and network sockets. Backpressure means that data are received at a higher rate than they can be processed during a temporary load spike. Many everyday situations can cause backpressure, especially for socket-based systems that are designed to handle a huge number of distributed nodes, like fleet management systems. On average, at least 1000 nodes are connected simultaneously in basic-level fleet management systems, and so adding an inspection process at this layer will cause a huge load and result in functioning for thread based systems as each NMEA packet will be defragmented and rephrased to update the date value after fixing the week number with the correct value. Such a process means applying the mathematical model proposed in this research to fix such an issue.

#### 2.2.3. Database Level Trigger Solution

One of the key features provided within the internal design of the database can be used effectively to activate a specific event inside the database as a response to a specific action. A database trigger which works as an internal procedural statement can accomplish such a task by calling for a specific procedural statement which will finally update the data in terms of time and can be used to apply mathematical operations. Using database triggers is not limited to process in which mean it is used as a respond to the data manipulation process. INSERT and UPDATE actions are the two main statements used within trigger operations. Accordingly, if you wanted to edit any data before inserting it into the table, you simply need to write a trigger script which will be activated if a specific condition triggers it [14].

Using triggers is highly efficient for processes related to data manipulation. For example if you have a vehicle management system and need to check the integrity of the data before it is entered into the database, you can write a trigger to check the inserted data and even process it, if needed. Such data manipulation is mostly related to time and date constraints. Writing triggers is a sensitive matter in terms of database engineering, as you need to understand the different types of triggers and the effects of this over the database engine. Many programmers only devote attention to the trigger itself as a task without understanding the wide range of differences for different forms of triggers and the effects of programming triggers. For example, writing a trigger inside a database with millions of records that is related to a specific table that has a significant amount of data indexing, trees and special data processes needs to be very precise and accurate in order to protect the database from performance-related issues [15].

For example, starting multiple triggers at the same time that are related to the same SQL statement or record need to be handled with skill in order to save the SQL statement passing process. This will result in highly effective replies in terms of the data size and time needed, which also represents a huge enhancement over the whole network.

**Figure 4:**
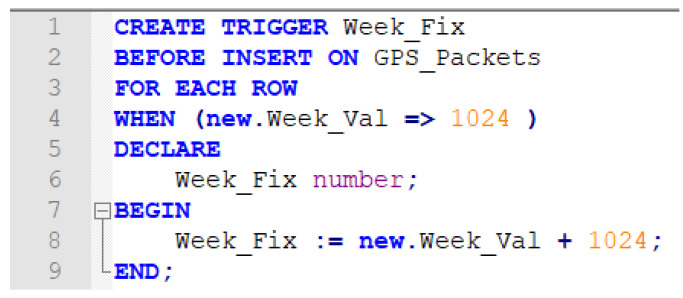
Simple trigger creating process over a trial database used to store and handle GPS NMEA packets, a simple process is applied before any data are entered into the database for processing.

As demonstrated in Figure 4, we use a database trigger to handle the data editing process before entering data into the main table that handles the final data used inside the fleet management system. In Figure 4, we simulate the process of creating a trigger that only adds 1024 weeks to the original data that were passed on from the device.

## 3. Results

After analyzing the GPS rollover problem, which is basically a mathematical problem, we have found that solving this by updating the hardware is very expensive in terms of time and equipment. Device replacement, in most of the cases, represents a huge cost. For example, in Fleet Management Systems (FMS), you need a specialized technician to visit each vehicle and obtain a device replacement which is a process that includes vehicle assembling and de-assembling. From the data provided by Traklink, we have selected a client who has a rental office with a fleet of 500 rental cars, all of which are equipped with a GPS device that uses an Atmel AVR XMEGA processor with 256 KB of Flash Memory, 16KB SRAM and operates at 32 MHz frequency. Three types of solutions were tested. The first solution that was tested was updating the firmware using the over-the-air (OTA) concept, which requires upgrading the program on the main firmware using another processing system that is used specifically for OTA processes. As shown in Figure 3, firmware upgrade-based solutions, which are applied using a C code that accomplishes the complement concept by 0.0013 s, resulted in almost zero delay for the data between the device and the main server that received the data sent from the GPS devices. In terms of the cost, the development of such a solution requires very specific equipment regarding the development and chips firmware upgrade, and, as such, after calculating the cost for the whole fleet we found that each unit cost almost 10 USD to be firmware updated.

Regarding data handler solution, it is basically achieved by software working at the level of the application layer within the OSI layer and receiving a packet using TCP or UDP. The packet is then analyzed in order to detect the value of the week’s number within the CNAV message. Following this, the time offset needed is calculated according to the equations explained in Section 2.1 of this paper. As such, time calculations, in this case, are related to server performance hardware and, after testing this solution over Intel Xeon E5 2630v4 and a multi-threaded socket handler working over TCP, we have found that each packet needs almost 0.164 s to be proceed using the complements equations explained in Section 2.1. In addition, the cost of development in the market is almost 3 USD for each unit. Regarding the delay, a 15% delay was calculated from the time needed to access the server.

Regarding the database trigger solution—which is the easiest solution in terms of implementation—we noticed that applying the complement equations using database triggers can fix the incorrect week number that is sent from the device. After the device sends the data to the server using TCP protocol, a TCP handler will send these data to a specific database that is used by the FMS system. After entering these data into the database table, the data will be pre-proceed using a database trigger that applies the complement equations that are represented and the data will then be entered into the table. In terms of performance, and after applying this within MySQL, 1.247 s were needed for each transaction from the time the packet sent until the moment it was entered into the table. The delay ration for such a solution is not accepted for specific time critical solutions, as 38% of the delay from the original time is not accepted in systems like the Advanced Driver Assistance Systems (ADAS). In term of cost, this solution represents the cheapest option.

**Figure 5:**
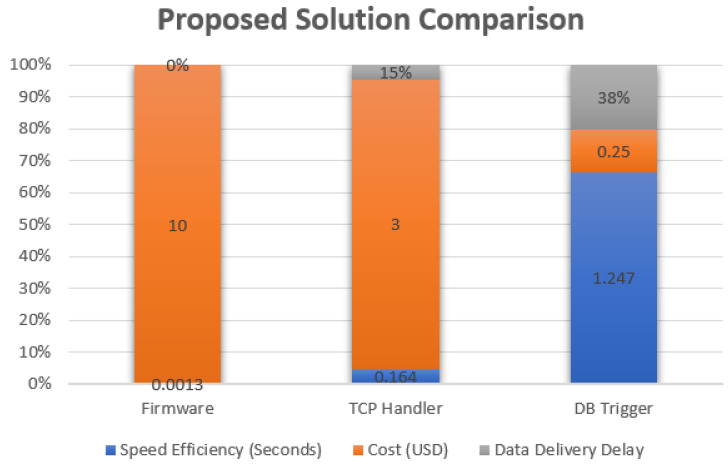
Proposed solutions comparison in term of speed, cost and data delivery delay.

After analyzing the results, we found that the most effective method in terms of cost is the DB trigger method, followed by the TCP Handler method and, finally, the Firmware upgrade, which represented the most expensive solution as shown in Figure 5. In terms of efficiency, we found that the firmware is the most effective and efficient solution, followed by the TCP handler method and, finally, the DB trigger method.

## 4. Discussion

According to the results, applying the complement solution is mathematically correct and applicable. Regarding the application mechanism, this is an open option, but it is clear that upgrading the firmware is the best solution in terms of efficiency and delay condition for data communication, especially in applications for which hard real-time is needed. However, if the application is not related to real-time limitations, it is appropriate to use other available solutions to implement the proposed mathematical solution at the lowest possible cost. Regarding firmware efficiency, it does represent the most expensive solution as it does require connecting to every unit to upgrade the firmware. Updating the firmware is an expensive option and requires special programming skills and a thorough knowledge of embedded systems. Updating the TCP Handler is not cheap and not expensive, but also causes delays due to the forwarding process. Finally, using the DB trigger mechanism will cause a significant time delays, which can have ramifications especially for those application which have hard real-time constraints.

## 5. Conclusions

GPS week number issue is a design flaw in CNAV protocol that limits the space of the week identification variable to a specific number, causing unexpected behavior in the subsystems that use GPS raw data for processing. GPS leap year differences and time offsets between UTC, GMT, and the GPS leap year should be taken into consideration when building a mathematical model to handle mathematical distortions caused by week counter resets that result in incorrect dates. Data transmitted from a GPS unit containing an incorrect week number was passed through several stages so that we could apply our proposed mathematical model at each stage. Implementing the proposed solution varied from stage to stage in terms of cost, efficiency, and delay time. Handling the data from inside the device by updating the firmware will solve the issue, but the complexity and cost are too high. Database triggers are the most efficient solution that can be applied to overcome such problems and maintain data integrity in terms of cost, while using firmware solution will provide the most efficient solution with almost no delays. After applying the three proposed solutions, we found that they varied in terms of cost, efficiency, and delay. As such, the solution needs to be chosen depending on the application.

## Figures and Tables

**Table 1:** GPS week number rollover epoch occurrences starting from the first lunch of GPS system.

Week Cycle	Starting Point	End of Cycle
1	6 January 1980 (0)	21 August 1999 (1024)
2	22 August 1999 (1024)	6 April 2019 (2047)
3	7 April 2019 (2047)	20 November 2038 (3071)
4	21 November 2038 (3071)	6 January 2137 (8192)

**Table 2:** Real dataset from different models of GPS units and effects of GPS week number rollover.

Model	Corrected Date	Wrong Date
Bofan PT95	12 December 2010	28 April 1991
Benway GT02B	11 August 2013	26 December 1993
cTrack	17 September 2017	1 February 1998
GN77C1	19 August 2018	3 January 1999
Teltonika FM1100	16 September 2018	31 January 1999
GN78	17 March 2019	1 August 1999

**Table 3:** Mathematical symbols used in mathematical representation.

Model	Description
STs	Timestamp for over rolled date
Ut	Unix Timestamp epoch at 1 January1970 00:00:00
Gs	GPS Timestamp epoch at 6 January 1980 00:00:00
UGs	Offset between Ut and Gs 315,964,800 s
Ws	Number of seconds in 1 week 604,800 s
OTs	Over rolled date after applying mathematical model calculations
Td	Average offset between International Atomic Time (TAI) and UTC

## Data Availability

The data presented in this study are available on request from the corresponding author. The data are not publicly available due to the restriction of local law and government policy.
